# The angiogenic genes predict prognosis and immune characteristics in esophageal squamous cell carcinoma: Evidence from multi-omics and experimental verification

**DOI:** 10.3389/fonc.2022.961634

**Published:** 2022-09-08

**Authors:** Shuaiyuan Wang, Yinghao Liang, Jiaxin Zhang, Wenjia Wang, Yichen Hong, Miaomiao Sun, Jiao Shu, Kuisheng Chen

**Affiliations:** ^1^ Department of Pathology, The First Affiliated Hospital of Zhengzhou University, Zhengzhou, China; ^2^ Academy of Medical Sciences, Zhengzhou University, Zhengzhou, China; ^3^ Henan Key Laboratory of Tumor Pathology, Zhengzhou University, Zhengzhou, China; ^4^ Henan Institute of Medical and Pharmaceutical Sciences, Zhengzhou University, Zhengzhou, China; ^5^ Department of Oncology, The First Affiliated Hospital of Zhengzhou University, Zhengzhou, China

**Keywords:** esophageal squamous cell carcinoma, angiogenesis, multi-omics, TME, immunotherapy, prognosis

## Abstract

Esophageal squamous cell carcinomas (ESCC) is an aggressive disease with five-year overall survival (OS) <15%. The main cause is metastasis rather than local tumor, and angiogenesis plays an important role. Angiogenesis has a significant impact on tumor metastasis, treatment and prognosis. However, the expression pattern of angiogenic genes, its effect on treatment and its relationship with prognosis in ESCC have not been systematically reported. We performed the first and most comprehensive multi-omics analysis of angiogenic genes in patients with ESCC and identified four angiogenic phenotypes that vary in outcome, tumor characteristics, and immune landscape. These subtypes provide not only patient outcomes but also key information that will help to identify immune blocking therapy. In addition, angiogenesis intensity score (AIS) was proposed to quantify tumor angiogenesis ability, and its accuracy as a predictor of prognosis and immunotherapy was verified by external cohort and corresponding cell lines. Our study provides clinicians with guidance for individualized immune checkpoint blocking therapy and anti-angiogenic therapy for ESCC.

## 1 Introduction

ESCC is a disease with a high incidence and high malignancy, of which >30% of patients have occur recurrence and metastasis (1). Most patients have insidious symptoms, leading to local infiltration and lymph node metastasis at the first time of diagnosis. Endoscopy indicates that early stage of ESCC can be accompanied by angiogenesis and morphological changes of microvessels (2). Angiogenesis can supply oxygen and nutrients for tumor, while providing opportunities for tumor cells to enter the circulation. Neocapillaries are more easily penetrated than mature vessels (3). In turn, tumors release and induce multiple angiogenic and anti-angiogenic factors, such as vascular endothelial growth factor (VEGF) and IL-8, which regulate the proliferation, migration, and apoptosis of endothelial cell (EC). Thus, angiogenesis plays an important role in growth and metastasis of many solid tumors (2–4).

Tumor vasculature is involved in various aspects of cancer activities, such as response to tumor microenvironment(TME), metabolism and drug resistance. Innate immune cells, as a component of the TME, can alter their phenotype that can release proangiogenic cytokines (5). ECs have been reported to become an important origin of cancer-associated fibroblasts, and transforming growth factor-*β* is responsible for such ECs conversion in endothelial-mesenchymal transition (6). Sterol regulatory element-binding proteins, transcription factors that maintain cellular lipid homeostasis, are crucial for regulating angiogenesis in response to VEGF (7), which present metabolic characteristic during angiogenesis. Drug inhibition of angiogenesis is an area of intense research and more than 300 angiogenesis inhibitors have been discovered (8). Angiogenesis inhibitors can be divided into direct and indirect inhibitors. The direct inhibitors target vascular ECs, such as endostatin, and indirect inhibitors disturb the pro-angiogenic communication between tumor, stroma, and ECs, such as epidermal growth factor receptor inhibitors and vascular endothelial growth factor receptor antibodies (8). Nowadays, more studies have shown that the combination of angiogenesis inhibitors that target different pathways may be more effective than single agents.

The genomic comprehensive analyses of ESCC have been sequenced recently, and researchers reported numerous key genomic aberrations, including mutations, amplifications or deletions (9). ESCC is a disease with both inter- and intra-tumor heterogeneity (9, 10), the diversity in the molecular profile of ESCC governs the tumor development including angiogenesis. Several genes, such as PLCE1, AKIP1 and Sox2, have been proved to regulate angiogenesis in ESCC (2, 11, 12), and blockade of the NF-*κβ* pathway can suppresses angiogenesis (13, 14). Therefore, it is important to discover the major genes that regulate angiogenesis in ESCC and their association with other cancer activities.

Here, we perform the genetic and epigenetic studies of angiogenesis in ESCC for the first time. Firstly, a multigene model to classify ESCC using angiogenic genes expression profiling is built based on The Cancer Genome Atlas (TCGA) and Gene Expression Omnibus (GEO). Then we analyze the prognosis and other cancer activities among different subtypes and these results are validated *in vitro* finally.

## 2 Materials and methods

### 2.1 Datasets

In this study, we downloaded two data sets, GSE53624 and GSE53625, from GEO database. Among them, GSE53624 data set was composed of 119 ESCC samples and 119 adjacent, matching non-tumor tissues; the GSE53625 dataset consisted of 179 ESCC samples and 179 adjacent, matching non-tumor tissues. In addition, RNA-seq data and clinical information of 78 ESCC patients were downloaded from TCGA. Somatic mutation data, copy number variation (CNV) and methylation data were obtained from TCGA. Because TCGA had multi-omics data, it was used for multi-omics analysis. ESCC patients with GSE53625 were classified as the training cohort, and patients within GSE53624 were used as the external validation cohort. The clinical characteristics of all patients were shown in **Table 1**. In order to make the gene expression profiling comparable between different platforms, the Trans Per Million values of RNA-Seq, robust multichip analysis processed values of miacroarry. The qRT-PCR data were log2 transformed and then normalized with the scale method by using the limma package in R. The “maftoofs” software package in R analyzes the mutation profiles and the “GenVisR” software package visualize the results. The CNV is defined as the total number of genes whose copy number changes in each sample, and the results are visualized using the “RCircos” software package in R. Gene methylation level is estimated by the average beta value of specific probe. Tumor related transcription factors (TF) were obtained from Cistrome cancer database.

**Table 1:** Clinical characteristics of the all patients.

Characteristics	GSE53625 (n=179)	GSE53624 (n=119)	TCGA (n=78)
	NO. (%)	NO. (%)	NO. (%)
Age (y)			
< 65	127 (70.9)	85 (71.4)	85 (71.4)
≥ 65	52 (29.1)	34 (28.6)	18 (23.1)
Gender			
female	33 (18.4)	21 (17.6)	12 (16.5)
male	146 (81.6)	98 (82.4)	66 (83.5)
Grade			
poorly	49 (27.4)	32 (26.9)	–
moderately	98 (54.7)	64 (53.8)	–
well	32 (17.9)	23 (19.3)	–
Stage			
I-II	87 (48.6)	53 (44.5)	54 (68.4)
III-IV	92(51.4)	66 (55.5)	24 (31.6)
Survive state			
alive	73 (40.8)	46 (38.7)	56 (70.9)
dead	106 (59.2)	73 (61.3)	22 (29.1)

### 2.2 Gene set variation analysis (GSVA)

To assess different tumor characteristics including angiogenesis, we downloaded the Hallmark gene set from the Molecular Signature Database (MSigDB). In order to study the potential mechanism of different subtypes, twelve canonical biological processes (15) are also assessed, including (1) DNA damage repair; (2) TGF-*β* response signature (Pan-F-TBRS); (3) antigen processing machinery; (4) immune checkpoint; (5) FGFR3-related gene; (6) cell cycle; (7) DNA replication; (8) nucleotide excision repair; (9) homologous recombination; (10) mismatch repair; (11) epithelial-mesenchymal transformation (EMT) markers, EMT1, EMT2 and EMT3; (12) WNT pathway. Additionally, we downloaded 10 canonical cancer pathways from the study of Sanchez-Vega et al. (16). The differences of scores between different phenotypes were analyzed by limma package in R. All gene sets were analyzed by ssGSVA package (17).

### 2.3 TME

Using the expression data, the matrix and immune components in each sample were scored by ESTIMAT algorithm, and the total score was calculated to explore the microenvironment differences between different groups. At the same time, the CIBERSORT algorithm was used to evaluate the proportion of twenty-two kinds of immune cells in each sample to study the difference of immune cell infiltration.

### 2.4 Quantitative real-time PCR

According to the description of the Japanese Collection of Research Bioresources (JCRB) cell bank and related literature, TE1 is representative of cell lines derived from orthotopic tumors (18–20), whereas EC9706 is representative of cell lines derived from metastatic tumor (21–23). So TE1 and EC9706 were selected as our research subjects. Het-1A, which is the normal esophageal epithelial cell line are reference (all obtained from the Institute of oncology, Chinese Medical College, Shanghai, China). To validate the accuracy of the model in predicting high- and low- risks, RT-qPCR was performed using SYBR qPCR Master Mix (Vazyme). RT-qPCR primer sequences: PTK2 F: 5’CTACAGCCTTATGACGAAATGC 3’, R: 5’ CTTCTCTTCCTCCAGGATTGTG 3’; TIMP1 F: 5’CATCACTACCTGCAGTTTTGTG 3’, R: 5’ TGGATAAACAGGGAAAACCTGT 3’; GAPDH F: 5’GAAGGTGAAGGTCGAGAGTCA 3’; R: 5’AATGAAGGGGTCATTGATGG 3’.The gene expression level of Het-1A was normalized by 2 *^ΔΔCT^*, which was used to quantify the expression levels of individual genes, to calculate a score for cells of different degree of malignancy.

### 2.5 Cell colony formation assay

The cells were seeded into a 24-well plate at a density of 400 cells/well, and gently shaken to make the cells evenly distributed. Each group had three replicates. The medium was changed every 3 days, and cell colonies were observed after 7 days. The cells were washed with PBS, fixed with 4% paraformaldehyde for 30 minutes at room temperature, and stained with 0.5% crystal violet for 20 minutes. The stains were carefully washed with pure water and then dried. Each well was photographed and counted.

### 2.6 Statistical analysis

The independent Student’s t test for continuous data and the *χ* ^2^ test for categorical data were utilized for pairwise comparisons between groups. Comparisons of categorical and non-normally distributed variables between two groups were performed using the Mann-Whitney U test and Kruskal-Wallis H test for multiple groups. The spearman correlation test assessed the correlation between normally distributed variables. A *P*-value <0.05 and |*correlation – coefficient(R)*| > 0.3| were considered significantly correlated. The threshold for statistical analysis in this study was set at A two-tailed *P*-value < 0.05. TFs of angiogenic genes were analyzed in combination with human TF information (NetworkAnalyst, http://www.networkanalyst.ca) and visualized using Cytoscape software (24). Then Cytoscape’s plugin, iRegulon, was used to predict TF regulatory networks.

## 3 Results

### 3.1 Association of angiogenesis with tumor characteristics and prognosis

**Figure 1:**
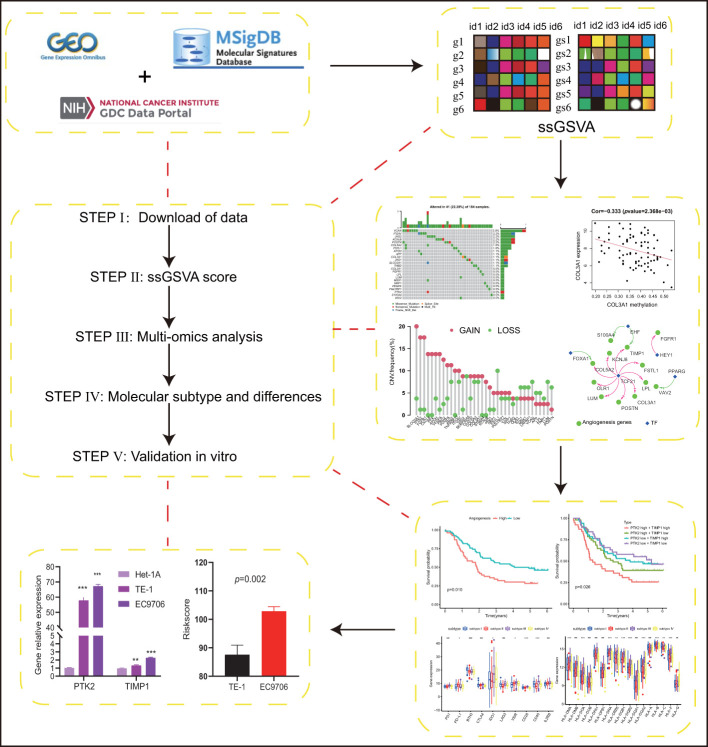
Schematic illustration of our research,subtypes. (p values were showed as: *p < 0.05; **p < 0.01; ***p < 0.001. ****p < 0.0001).

**Figure 2:**
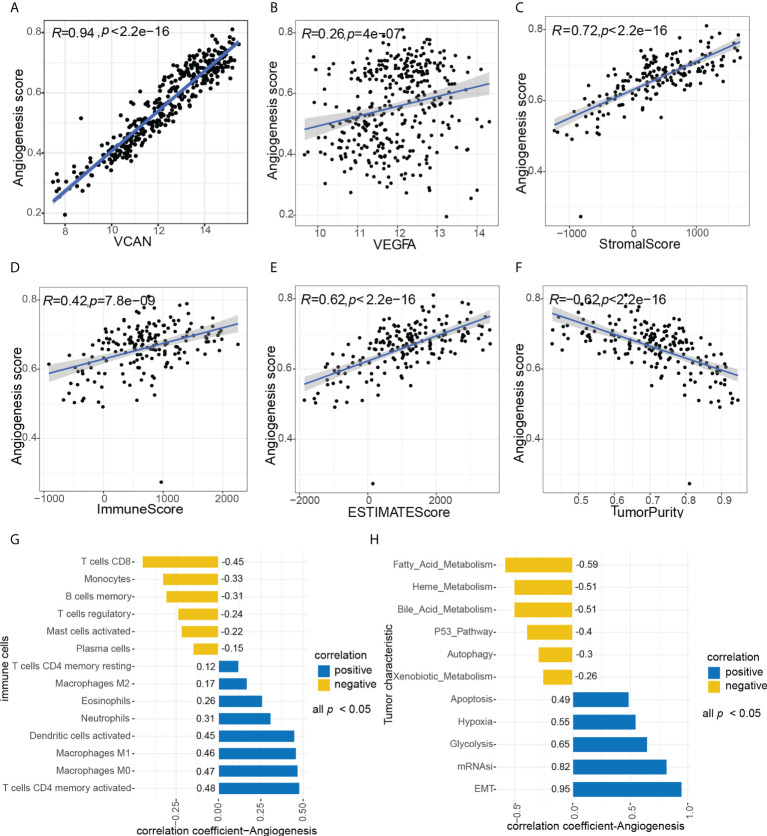
Association between angiogenesis with tumor characteristics. **(A**, **B)** Association between angiogenesis score and representative angiogenesis genes, A (VCAN), B(VEGFA). **(C–F)** Correlation of angiogenesis score with TME and tumor purity, C (StromalScore), D (ImmuneScore), E (ESTIMATEScore), F (TumorPurity). **(H)** Association between angiogenesis score and immune cells, all p value < 0.05. **(G)** Association between angiogenesis score and tumor characteristics, all p value < 0.05.

**Figure 3:**
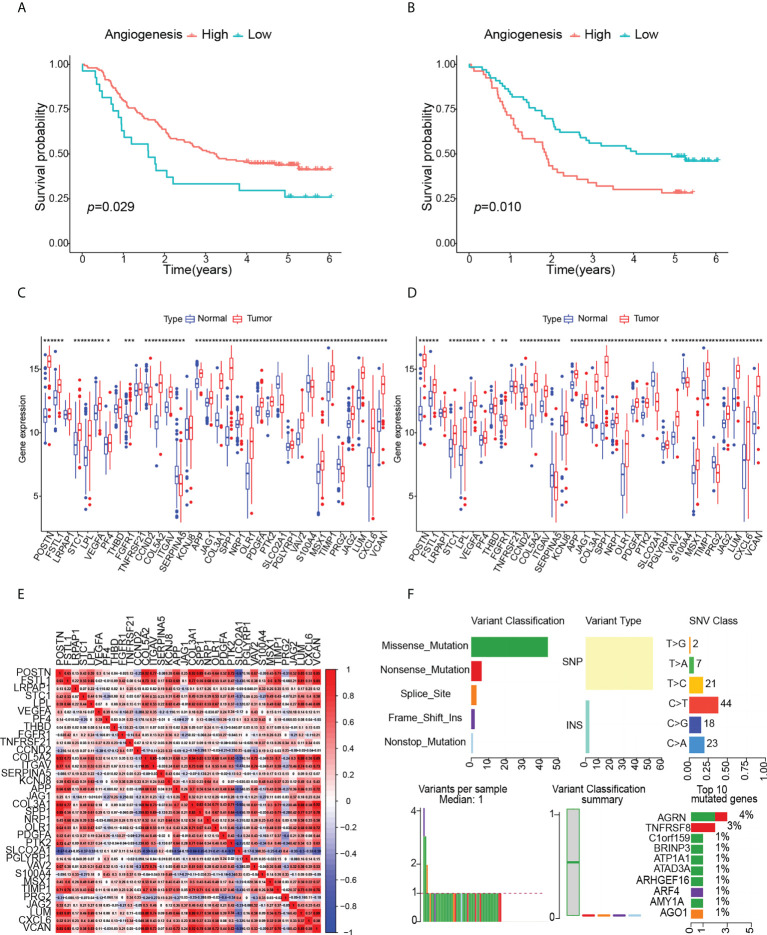
Effects of angiogenesis on prognosis and analysis of angiogenesis genes, (p values were showed as: *p < 0.05; **p < 0.01; ***p < 0.001). **(A)** Kaplan-Meier curve of OS for high- angiogenesis and low- angiogenesis groups in GSE53625. **(B)** Kaplan-Meier curve of OS for high- angiogenesis and low- angiogenesis groups in GSE53624. **(C)** Expression levels of angiogenesis genes in GSE53625. **(D)** Expression levels of angiogenesis genes in GSE53624. **(E)** Correlation between angiogenesis genes. **(F)** Landscape of somatic mutation in ESCC patients.

The overall workflow of this study was shown in **Figure 1**. ESCC scores for tumor characteristics including angiogenesis were calculated using ssGSVA. To evaluate the accuracy of the angiogenesis score, we selected several representative angiogenic genes and observed good correlation between the angiogenesis score and the expression levels of VCAN and VEGFA (**Figures 2A, B**). The strong correlation of the angiogenesis score with the TME and tumor purity further demonstrated the importance of angiogenesis and the reliability of the score (**Figures 2C–F**). The relationships between immune cells and angiogenesis were also investigated. CD8 ^+^ T cells and memory B cells inhibited angiogenesis, and macrophages and activated dendritic cells greatly enhanced angiogenesis (**Figure 2G**). Tumor angiogenesis underlies tumorigenesis and progression. We found that angiogenesis is tightly associated with distinct features of ESCC tumor (**Figure 2H**). More importantly, K-M survival analysis showed that angiogenesis had a significant impact on OS in ESCC patients, which was validated in another dataset (**Figures 3A, B**).

### 3.2 Multi-omics analysis of angiogenic genes

To identify the multi-omics map and expression drivers of angiogenic genes in ESCC, we firstly compared the expression changes of angiogenic genes between paired normal and ESCC samples. The results showed that the expression of angiogenic genes was highly heterogeneous between normal and ESCC tissues, and the mRNA levels of most angiogenic genes were significantly increased in tumor tissues (**Figures 3C, D**), which further indicated the important role of angiogenesis in ESCC. We calculated the angiogenic intergenic spearman correlation in ESCC to explore the intergenic relationships. The results suggested that positive correlations were more frequent than negative correlations. In particular, the expression of SLCO2A1 was negatively correlated with other angiogenic genes (**Figure 3E**). Synergy among different angiogenic genes may be important for the regulation of angiogenesis in individual tumors. It is important to systematically analyze the effects of angiogenic genes on ESCC patients.

**Figure 4:**
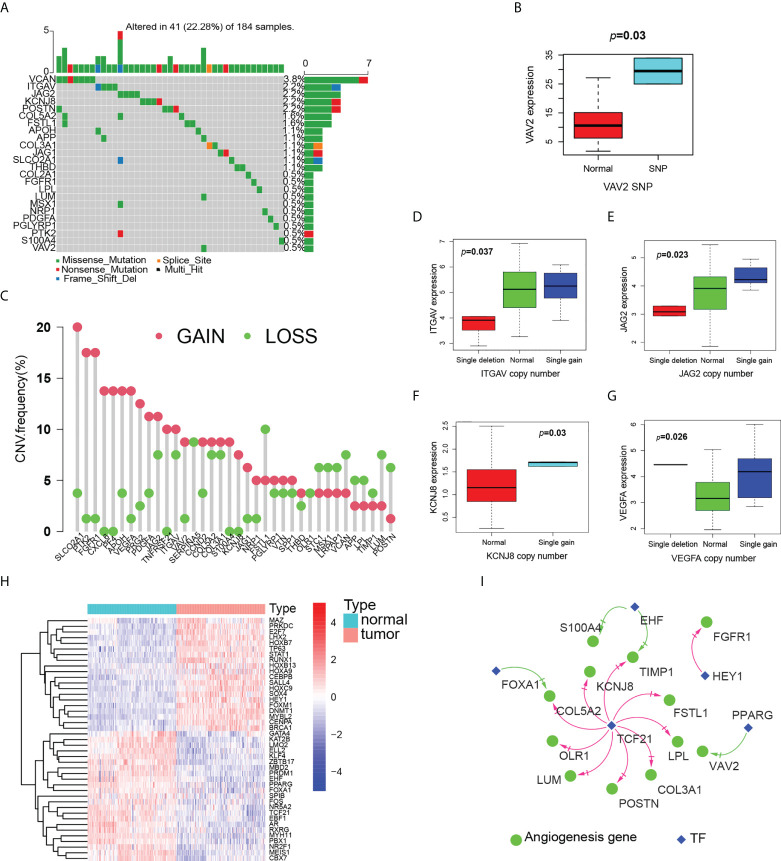
Multiomics analysis of angiogenesis genes. **(A)** Landscape of mutation of angiogenesis genes in ESCC patients. **(B)** Boxplot of the relationship between VAV2 expression and SNP. **(C)** Landscape of CNV of angiogenesis genes in ESCC patients. **(D–G)** Boxplot of the relationship between expression of angiogenesis genes and CNV, D(ITGAV), E(JAG2), F(KCNJ8),G(VEGFA). **(H)** Heatmap (blue: low expression level; red: high expression level) of the TF with p < 0.05 between the normal and the tumor tissues. **(I)** The correlation network of expression between angiogenesis genes and TF. (red line: positive correlation; blue line: negative correlation).

Next, we comprehensively analyzed the relationships among somatic mutations, CNVs, methylation, transcription factors and angiogenic gene expression. ESCC somatic mutations were dominated by Single Nucleotide Polymorphism (SNP) and involved six types of single nucleotide variations (SNV), of which C > T was predominant and the missense mutations were the most common, including angiogenic genes. Among the top ten mutated genes, ATP1A1, C1orf159 have been reported to be associated with ESCC development, and other novel mutated genes we identified, such as AGRN, TNFRSF8, BRINP3, ATAD3A, ARHGEF16, ARF4, AMY1A, AGO1, have been studied in some diseases, but lacking a study with ESCC (**Figure 3F**). The SNP landscape of angiogenesis genes was shown in **Figure 4A**. In addition, we also found the correlation between VAV2 mutation and expression (**Figure 4B**).

We then examined CNV of angiogenic genes and found that SLCO2A1, PTK2, FGFR1, CXCL6, PF4 and VEGFA had widespread frequency of CNV gain, while FSTL1, VCAN, LUM and POSTN were CNV loss (**Figure 4C**). CNV generally affects gene expression. We further explored to find that angiogenic genes with CNV gains were more frequent in ESCC and highly likely to regulate the expression of ITGAV, JAG2 and KCNJ8 mRNA (**Figures 4D–F**). Some gene expression is up-regulated, but CNV is loss, such as VEGFA (**Figure 4G**). These genes may be influenced by other factors at the same time.

Ultimately, we identified nine angiogenic genes that may be affected by methylation (**Supplementary Figure 1**). In particular, ITGAV is co-driven by both CNV and methylation. Genetic and epigenetic alterations that drive ITGAV expression changes lead to ESCC development. Further, we compared the expression of TF between normal esophagus and ESCC, and there were obvious differences between the two (**Figure 4H**). By them, we constructed transcriptional regulatory network with fourth edges and five nodes (**Figure 4I**). Notably, TCF21 was predicted to regulate most angiogenic genes, such as POSTN, FSTL1, LPL, COL5A2, KCNJ8, COL3A1, OLR1, TIMP1 and LUM. The value of TCF21 in ESCC has been reported, but there is no mechanistic study, and our findings provide ideas for the mechanism of TCF21 in ESCC.

### 3.3 ESCC typing based on angiogenesis

**Figure 5:**
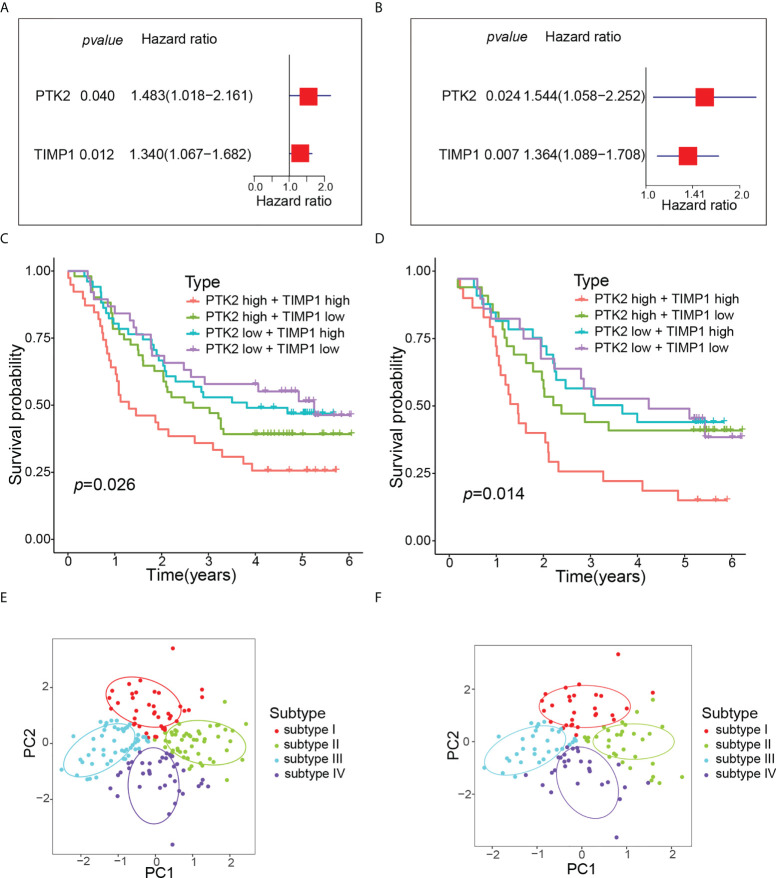
Molecular typing of ESCC patients. **(A)** Univariate Cox regression analysis of OS according to angiogenesis genes expression in GSE53625. **(B)** Multivariate Cox regression analysis of OS according to angiogenesis genes expression in GSE53625. **(C)** Kaplan-Meier curve of OS for four subtypes in GSE53625. **(D)** Kaplan-Meier curve of OS for four subtypes in GSE53624. **(E)** PCA revealed that all ESCC patients were well divided into four subtypes in GSE53625. **(F)** PCA revealed that all ESCC patients were also well divided into four subtypes in GSE53624.

**Figure 6:**
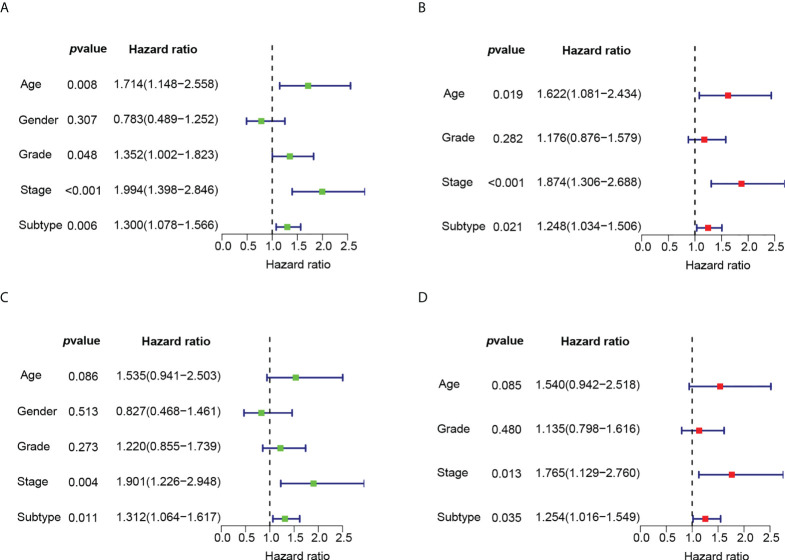
The univariate and multivariate Cox regression analyses of OS according to subtypes. **(A)** Univariate Cox regression analysis of OS according to subtypes in GSE53625. **(B)** Multivariate Cox regression analysis of OS according to subtypes in GSE53625. **(C)** Univariate Cox regression analysis of OS according to subtypes in GSE53624. **(D)** Multivariate Cox regression analysis of OS according to subtypes in GSE53624.

Univariate Cox regression analysis showed that PTK2 hazard ratio (HR) was 1.483 (95% confidence interval [CI]: 1.018-2.161) and TIMP1 HR was 1.340 (95% CI: 1.067-1.682), which were risk factors for OS in ESCC patients (**Figure 5A**). The results of multivariate analysis still showed that PTK2 and TIMP1 were risk factors for OS of ESCC patients (**Figure 5B**). According to the median expression values of PTK2 and TIMP1, ESCC patients were classified into subtype I: PTK2 *^high^* + TIMP1 *^high^* ; subtype II: PTK2*^high^* + TIMP1 *^low^* ; subtype III: PTK2 *^low^* + TIMP1*^high^* ; subtype IV: PTK2*^low^* + TIMP1 *^low^* We observed significant OS differences among the four subtypes (**Figure 5C**). This result was verified in GSE53624 (**Figure 5D**). Based on angiogenesis, Principal component analysis (PCA) compared the differences among the four subtypes, and the results revealed that the different subtypes were distributed in different directions (**Figures 5E, F**). This typing method could correctly classify ESCC patients into four subtypes. The univariate and multivariate Cox regression analyses further indicated that subtypes could independently predict the outcome of ESCC patients (**Figures 6A–D**).

### 3.4 TME differences among ESCC subtypes

**Figure 7:**
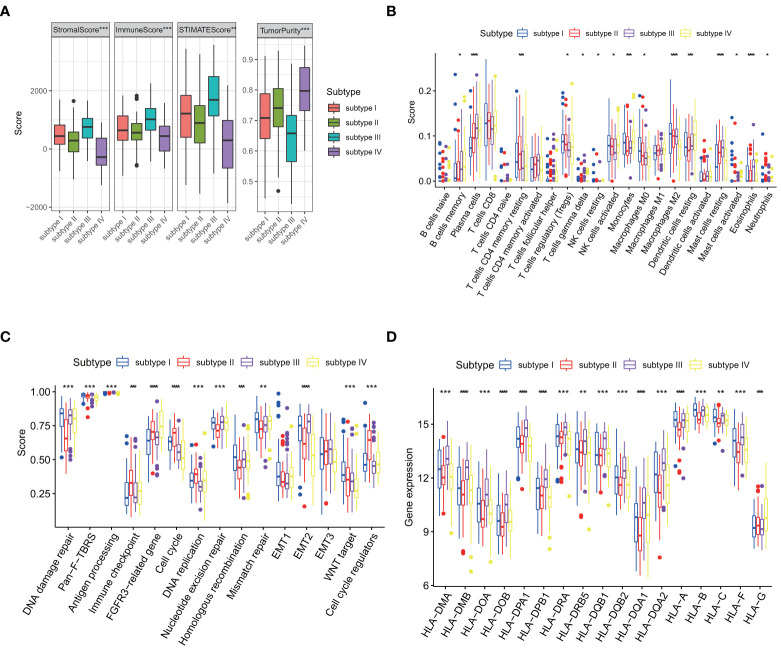
Comparison of four subtypes.(p values were showed as: *p < 0.05; **p < 0.01; ***p < 0.001. ****p < 0.0001). **(A)** Differences in TME and tumor purity among four subtypes in GSE53625. **(B)** Differences in immune cells among four subtypes in GSE53625. **(C)** Differences in core biological pathways among four subtypes in GSE53625. **(D)** Differences in HLA gene expression among four subtypes in GSE53625.

Recent studies have shown that the tumor immune microenvironment (TIME) plays a key role in cancer prognosis and treatment response (25). We compared the ImmuneScore, StromalScore, EstimateScore and TumorPurity of four subtypes. The results showed that the TME and TumorPurity were significantly different among four subtypes (**Figure 7A**). In addition, the immune cell composition was also significantly different among four subtypes (**Figure 7B**). Compared with subtype IV, subtype I had fewer activated NK cells and B cells, but more resting NK cells, resting T cells and M2 macrophages, suggesting that one of the reasons for the worst prognosis of subtype I may be the lack of immuneactivating cells and the increase of immunosuppressive cells. Both activated and resting NK cells were higher in subtype II than subtype III, whereas adaptive immunity, such as B cells, and inflammatory cells, such as neutrophils, were higher in subtype III than in subtype II. Then We hypothesized that subtype III activated adaptive immunity.

We also found that biological process disorder may mediate poor prognosis. In subtype I, Homologous recombination, Mismatch repair and WNT target significantly higher than the other three subtypes. FGFR3-related pathways, Cell cycle, DNA replication and Cell cycle regulators are enhanced in subtype II. Pan-fibroblast transforming growth factor-*β* response (Pan-F-TBRS), Antigen processing pathway and Epithelial-mesenchymal transition 2 (EMT2) are enhanced in subtype III. The activation of the DNA damage repair and nucleotide excision repair pathways may cause subtype III to have longer OS than subtype II but shorter than subtype IV, with an unobserved imbalance in biological process of subtype IV (**Figure 7C**).

### 3.5 Immune landscape of ESCC subtypes

The mRNA levels of seventeen human leukocyte antigen (HLA) genes were significantly different among the four types (**Figure 7D**). The main function of MHC molecules is to bind and present antigenic peptides for recognition by CD **^+^** and CD4 **^+^** T cells. Earlier we hypothesized that immunity mediated the difference in prognosis between the four subtypes, once again validating our speculation.

**Figure 8:**
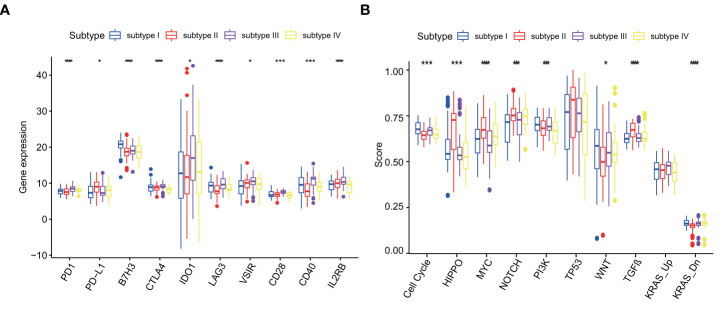
**(A)** Differences in immune checkpoint among four subtypes in GSE53625, p values were showed as: *p < 0.05; ***p < 0.001. ****p < 0.0001. **(B)** Differences in Cancer pathways among four subtypes in GSE53625, p values were showed as: *p < 0.05; ***p < 0.001. ****p < 0.0001.

The common 18 immune checkpoints (PD1, PD-L1, PD-L2, BTLA, B7H3, CTLA4, IDO1, LAG3, VSIR, TIM3, CD27, CD28, CD40, ICOS, IL2RB, GITR, OX40, 41BB) were matched to the genes measured in 257 ESCC patients. Finally, 10 immune checkpoints (PD1, PD-L1, B7H3, CTLA4, IDO1, LAG3, VSIR, CD28, CD40, IL2RB) were used for further analysis. Immune checkpoint expression varied greatly among four subtypes (**Figure 8A**). Compared with other subtypes, subtype III expressed more immune checkpoint stimulatory genes, such as CD28 and CD40, and inhibitory genes, such as CTLA4, IDO1, LAG3, VSIR, to achieve escape after immune activation.

### 3.6 Cancer pathways in ESCC subtypes

Different molecular subtypes have differential cancer pathway changes. Among ten recognized cancer pathways, nine have significant differences among four subtypes. There were four pathways in subtype I and subtype II that were significantly enhanced compared with other pathways. The four pathways of subtype I are Cell Cycle pathway, PI3K pathway, WNT pathway and KRAS-Down. The four pathways of subtype II are HIPPO, MYC, NOTCH and TGF-*β* . However, subtype III only has the highest change in KRAS-Up pathway and subtype IV lacks significantly changed pathways (**Figure 8B**).

### 3.7 AIS

**Figure 9:**
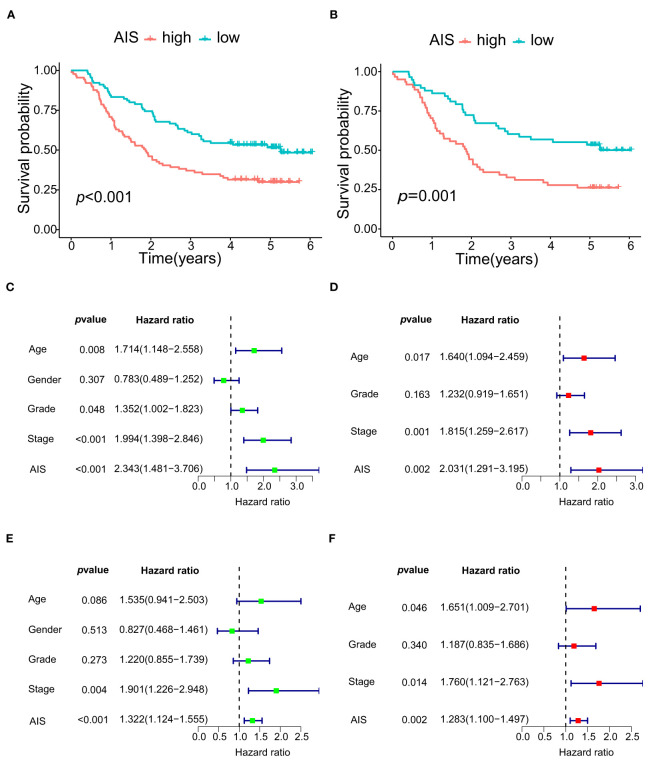
The survival and COX regression analyses of OS according to AIS. **(A)** Kaplan-Meier curve of OS for high- AIS and low- AIS groups in GSE53625. **(B)** Kaplan-Meier curve of OS for high- AIS and low- AIS groups in GSE53624. **(C)** Univariate Cox regression analysis of OS according to AIS in GSE53625. **(D)** Multivariate Cox regression analysis of OS according to AIS in GSE53625. **(E)** Univariate Cox regression analysis of OS according to AIS in GSE53624. **(F)** Multivariate Cox regression analysis of OS according to AIS in GSE53624.

Using PTK2 and TIMP1, we generated AIS to quantify angiogenesis. AIS =(EL of PTK2×1.48295148776218)+(EL of TIMP1× 1.33972979205648). As our predictions, patients in the low-AIS group showed significantly better prognosis than the high-AIS group, which was also verified in another data set (**Figures 9A, B**). The univariate and multivariate analysis showed that AIS as a risk factor can independently affect the prognosis of ESCC patients (**Figures 9C–F**).

### 3.8 Validation of signature *in vitro*

**Figure 10:**
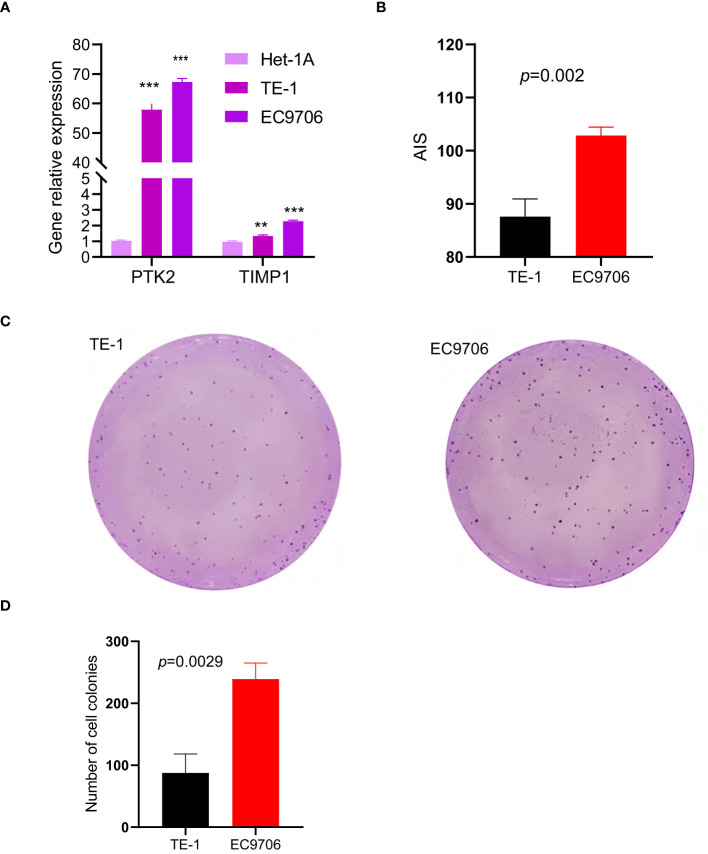
ESCC cell line experiments validate the predictive value of the two-gene signature in vitro, (p values were showed as: **p < 0.01; ***p < 0.001). **(A)** Expression levels of PTK2 and TIMP1 in two cancer cell lines and one normal cell line. **(B)** AIS of the two different risk cell lines calculated based on RT-qPCR results. **(C, D)** Plot of colony formation for two cancer cell lines.

The accuracy of the signature was verified in different risk cell lines. The results showed that compared with normal esophageal epithelial cell, both PTK2 and TIMP1 were highly expressed in ESCC cell lines (**Figure 10A**). What’s more, the higher the risk of cells is, the higher the AIS scores are (**Figure 10B**). In order to further evaluate the degree of malignancy of the two cancer cell lines and verify the reliability of our model, the cell colony formation assay was performed. As shown in **Figures 10C, D**, EC9706, which scored higher, formed more colonies, compared with TE-1. That proves all the results again.

## 4 Discussion

The vast majority of cancer-related deaths are caused by metastatic rather than local cancers (26), where angiogenesis plays an important role. Angiogenesis has been found to play an important role in the formation, progression and treatment of tumors, such as breast cancer and kidney cancer (27, 28). More evidence shows that angiogenesis is closely related to invasion, metastasis and survival of esophageal cancer (29, 30). Esophageal cancer is considered to be one of the main causes of cancer-related death. Traditional chemoradiotherapy has poor efficacy and severe side effects (31, 32). Anti-angiogenic therapy has gradually become a new therapy in recent years. However, more than half of patients cannot benefit from it and the five-year survival rate of esophageal cancer patients is still unsatisfactory. Therefore, it is necessary to study the expression patterns of angiogenic genes, biomarkers in ESCC and help make more accurate clinical decisions through multigene methods.

To explore meaningful prognostic indicators related to angiogenesis, we collected 376 patients with ESCC from three different cohorts in TCGA and GEO databases. For the first time, we systematically studied the major changes and possible driving factors of angiogenic genes in detail from the perspective of multi-omics. Then we constructed the prognostic signature related to two angiogenic genes, which were verified in the external datasets and further *in vitro*. It is worth mentioning that there are significant differences in OS, TME, immune and cancer pathways among different subtypes classified by this prognostic signature. Finally, we constructed a robust AIS to distinguish clinical high- or low-risk patients and guide clinical anti-angiogenic therapy and immunotherapy.

Some studies have shown that high expression of PTK2 and TIMP1 is associated with poor progression-free survival in patients with various cancers (33–36). But there is a lack of research in ESCC. Therefore, this is the first that ESCC patients are divided into four subtypes based on the expression levels of PTK2 and TIMP1. PTK2 protein tyrosine kinase is the alias of FAK, which is produced by PTK2 gene expression. In the process of tumor metastasis, the abnormal adhesion function between cells or between cells and matrix is of decisive significance, and Focal Adhesion Kinase (FAK) plays an important role in this function (37) There is growing evidence that truncation of FAK can reduce the mobility of cancer cells, thereby significantly reducing the risk of metastasis (38). The more activated PTK2, the higher the degree of involvement in cell adhesion and spread, which has guiding significance for the prognosis of multiple cancers, such as colorectal cancer, ovarian cancer and esophageal cancer (34, 39, 40). Li et al. (41) demonstrated that membrane metalloendopeptidase (MME) inhibits the transfer of ESCC by inhibiting FAK-RhoA signal axis. As reported by Tavora et al, depletion of EC-FAK reduced VEGF-induced Akt phosphorylation, inhibited angiogenesis and slowed tumor growth. Cancer-associated ECs also expressed more PTK2 and FAK protein as well as higher levels of phosphorylation (42, 43). Further studies revealed that reduced phosphorylation levels caused by PTK2 mutations attenuated endothelial cell proliferation, survival, migration and vascularization *in vitro* (44). Li Mengqing et al. (45) also demonstrated that, by inhibiting the FAK-RhoA signaling axis, MMEs inhibited the metastasis of ESCC. The relationship between PTK2 overexpression and metastasis has been reported. Therefore, PTK2 is an important driver molecule in tumor angiogenesis, and targeting PTK2 may be a promising therapeutic strategy for cancer.

The TIMP1 gene belongs to the TIMP gene family, which is a natural inhibitor of matrix metalloproteinases (MMPs). Normally, MMPs and TIMPs interact to keep the extracellular matrix (ECM) in homeostasis. However, there is evidence that if there is excessive TIMP1 in the tissue, it may activate other MMPs, such as MMP-3, or promote tumor invasion and metastasis through other modes of action (46, 47). The same tumor cells that secrete MMPs also synthesize TIMPs, the imbalance of MMP-9: TIMP1 makes tumor cells more invasive (36). TIMP1 may be a pluripotent protein with important functions for cancers. It has been shown that elevated TIMP1 expression in plasma and tumors of patients with breast (48), colorectal (49), gastric (50) and ovarian cancer (51), is associated with poor prognosis. Immunohistochemistry identified a concordance between high TIMP1 levels and a high rate of distant metastasis in breast cancer (52) and TIMP1, in serum of ovarian cancer patients with preoperative, is also associated with more aggressive tumor behavior (50). TIMP1 has been recognized as an anti-apoptotic protein that not only activates MMPs and regulates angiogenesis, but also inhibits apoptosis (53). Davidsen et al. (54) revealed that TIMP1 could inhibit chemotherapy-induced apoptosis by comparing TIMP1 gene deficiency variant and wild-type cells. Valsamma Abraham et al. (47) showed that PECAM-1, through PECAM-1-dependent homophilic ligand interaction, could induce TIMP1 release from the endothelium into the TME, leading to enhanced tumor cell proliferation. Mumtaz V. Rojiani et al. (55) demonstrated that overexpression of TIMP1 accelerated brain metastasis in lung cancer and observed a pro-angiogenic effect. The reason was speculated that MMPs would break down ECM and release anti-angiogenic factors, while TIMP1, through eliminating the action of matrix metallo proteinases, could promote angiogenesis (56). All these together with our findings prove the possibility of TIMP1 as a novel therapeutic target for ESCC and provide evidence for the accuracy of the prognostic signature we constructed.

Increasing evidence was reported that the TIME, including immune cells and cytokines, played a critical role in the induction of cancer and angiogenesis. Meanwhile, immune cells were also targets for angiogenesis inhibitors (57–59). Immunosuppressive cells not only secrete proinflammatory cytokines, such as IL-1, IL-2, IL-15 and IL-18, but also upregulate IL-8, an important chemokine in angiogenesis. These are strong angiogenic mediators and play an important role in regulating angiogenesis in TME (60–62). Macrophages undergo functional reprogramming and then are divided into two polarization states, M1 and M2. M2 macrophages promote tumor vascular development under hypoxic environment by inducing high expression of cytokines, including HIF-1, HIF-2, VEGF, basic fibroblast growth factor, IL-8 chemokines and lymphogenic factors. M1 macrophages promote inflammation and suppress tumors by suppressing angiogenesis and tissue remodeling (63). VEGFA/VEGFR2 or VEGFA pathway alone is associated with T-regs promoting angiogenesis in a variety of cancers (64). On the other hand, cancer-associated blood vessels are abnormal and tortuous, which can impair immune effector cells infiltration. TIME is shaped by changing tissue oxygen content, then altering immune cell composition and regulating various immune cells such as M2 macrophages, T-regs, MDSCs and related cytokines in TIME (65, 66). VEGF is an important angiogenic factor that is not only important for angiogenesis of tumors, but also, through binding to VEGFR on immune cells, upregulates the expression of immune checkpoints (PD-1, CTLA-4, TIM-3, LAG-3), thereby leading to the impairment of the function of effector T cells and T cells exhaustion (67). Studies have been reported that, in TME, hypoxia caused by abnormal tumor vasculature modulated the proportion of M2 macrophage population and decreased the proportion of M1 macrophage population (68).

Currently, it is widely recognized that tumors can be classified into three immune phenotypes: immune inflamed, immune excluded, or immune desert (69). The inflammatory phenotype shows the characteristics, such as high immune, inflammatory cell infiltration, high immune checkpoint expression and intact antigen status. Excluded types displayed properties related to the TGF-*β* signaling pathway, such as the presence of reactive stroma, myeloid-derived suppressor cells, and tumor angiogenesis. The immune desert type, which is characterized by lack of immune cell infiltration, lack of antigen presentation, and high tumor growth, increase fatty acid metabolism and WNT/*β* -Catenin signaling (70). We found that, among four subtypes of ESCC based on angiogenesis, subtype I conforms to immune desert characteristics, subtype II conforms to immune excluded, and subtype III and subtype IV belong to immune inflamed, especially subtype III. Sanjeev Mariathasan et al. found that inflamed tumors responded most strongly to checkpoint blocking (15), which is consistent with our findings. Our findings also provide ideas for immune checkpoint blocking therapy in ESCC.

This study has several strengths. Firstly, we systematically and comprehensively performed a multi-omics exploration of expression signatures and drivers of angiogenic genes in ESCC. Secondly, most of the current research and application of angiogenic genes are based on a single gene, which is one-sided. We focused on collecting all the angiogenic genes, constructing multigene prognostic signature, and reasonably dividing them into four subtypes to facilitate the formulation of clinical programs. Thirdly, our prognostic signature can independently assess the prognosis of ESCC patients and is closely related to the TME, immunity and tumor-related pathways. Fourthly, our findings are not only validated by external datasets, but also demonstrated by cell experiments *in vitro*. Importantly, immune checkpoints are significantly different in the four ESCC subtypes, which may lead to greater clinical benefits for immune checkpoint blocking therapy.

Anti-angiogenic therapy has been enduring and has shown clinical benefit in a variety of tumors, such as non-small cell lung cancer (71) chondrosarcomaa (72). Clinical research and data during decades have confirmed that anti-angiogenic therapy combined with PD-1/PD-L1 mAb immunotherapy can enhance therapeutic efficacy (73). However, in esophageal cancer, the clinical effects of anti-angiogenic therapy have been unsatisfactory. In clinical practice, the drug resistance of anti-angiogenic therapy, the lack of biomarkers for selecting potential patients and predicting effective response are the main reasons for the unsatisfactory efficacy of anti-angiogenic therapy in patients with ESCC. These are closely related to the responsiveness of the TME (74). Further development of biomarkers that can screen the dominant population, predict treatment efficacy, the combination forms of combination drugs and the mechanisms of resistance to anti-angiogenic therapy are the future directions. Among them, the relationship between TME and tumor angiogenesis is the research focus. We constructed four molecular subtypes based on angiogenic genes, generated AIS, and proposed potential biomarkers related to anti-angiogenic therapy. Our model can predict prognosis, help clinicians select ESCC patients most likely to benefit from anti-angiogenic therapy, guide clinicians in developing potentially effective treatment strategies for other ESCC patients and provide a basis for combination immunotherapy with anti-angiogenic therapy.

## Data availability statement

The datasets presented in this study can be found in online repositories. The names of the repository/repositories and accession number(s) can be found in the article/**Supplementary Material**.

## Author contributions

KC and SW designed the study and revised the manuscript. YL, JZ, and WW drafted the manuscript. SW, JS, and MS collected expression profile and clinical data. YH conducted cell experiments *in vitro*. SW and YL designed and prepared the figures. All authors read and approved the final manuscript.

## Funding

This study was supported by the Health Commission of Henan Province Innovative Talents Project [grant number 51282].

## Acknowledgments

All authors acknowledge the Gene Expression Omnibus (GEO) dataset, the Cancer Genome Atlas (TCGA) datasets and the Molecular Signature Database (MSigDB).

## Conflict of interest

The authors declare that the research was conducted in the absence of any commercial or financial relationships that could be construed as a potential conflict of interest.

## Publisher’s note

All claims expressed in this article are solely those of the authors and do not necessarily represent those of their affiliated organizations, or those of the publisher, the editors and the reviewers. Any product that may be evaluated in this article, or claim that may be made by its manufacturer, is not guaranteed or endorsed by the publisher.
